# *Weissella cibaria* riboflavin-overproducing and dextran-producing strains useful for the development of functional bread

**DOI:** 10.3389/fnut.2022.978831

**Published:** 2022-10-04

**Authors:** Annel M. Hernández-Alcántara, Rosana Chiva, María Luz Mohedano, Pasquale Russo, José Ángel Ruiz-Masó, Gloria del Solar, Giuseppe Spano, Mercedes Tamame, Paloma López

**Affiliations:** ^1^Departamento de Biotecnología Microbiana y de Plantas, Centro de Investigaciones Biológicas Margarita Salas, CSIC, Madrid, Spain; ^2^Instituto de Biología Funcional y Genómica, CSIC-Universidad de Salamanca, Salamanca, Spain; ^3^Department of Agriculture Food Natural Science Engineering, University of Foggia, Foggia, Italy

**Keywords:** lactic acid bacteria, *Weissella cibaria*, dextran, exopolysaccharide, riboflavin, vitamin B_2_, functional food, functional bread

## Abstract

This work describes a method for deriving riboflavin overproducing strains of *Weissella cibaria* by exposing three strains (BAL3C-5, BAL3C-7, and BAL3C-22) isolated from dough to increasing concentrations of roseoflavin. By this procedure, we selected one mutant overproducing strain from each parental strain (BAL3C-5 B2, BAL3C-7 B2, and BAL3C-22 B2, respectively). Quantification of dextran and riboflavin produced by the parental and mutant strains in a defined medium lacking riboflavin and polysaccharides confirmed that riboflavin was only overproduced by the mutant strains, whereas dextran production was similar in both mutant and parental strains. The molecular basis of the riboflavin overproduction by the mutants was determined by nucleotide sequencing of their *rib* operons, which encode the enzymes of the riboflavin biosynthetic pathway. We detected a unique mutation in each of the overproducing strains. These mutations, which map in the sensor domain (aptamer) of a regulatory element (the so-called FMN riboswitch) present in the 5’ untranslated region of the *rib* operon mRNA, appear to be responsible for the riboflavin-overproducing phenotype of the BAL3C-5 B2, BAL3C-7 B2, and BAL3C-22 B2 mutant strains. Furthermore, the molecular basis of dextran production by the six *W. cibaria* strains has been characterized by (*i*) the sequencing of their *dsr* genes encoding dextransucrases, which synthesize dextran using sucrose as substrate, and (*ii*) the detection of active Dsr proteins by zymograms. Finally, the parental and mutant strains were analyzed for *in situ* production of riboflavin and dextran during experimental bread making. The results indicate that the mutant strains were able to produce experimental wheat breads biofortified with both riboflavin and dextran and, therefore, may be useful for the manufacture of functional commercial breads.

## Introduction

Riboflavin (vitamin B_2_) is a water-soluble vitamin produced by plants and many microorganisms. Riboflavin is the precursor of flavin mononucleotide (FMN) and flavin adenine dinucleotide (FAD), both of which act as electron carriers in oxidation–reduction reactions, functioning as coenzymes for hundreds of FMN- or FAD-dependent enzymes called flavoproteins (1). Humans do not synthesize riboflavin, which, consequently, has to be obtained from the gut microbiota and the diet. Any excess of riboflavin is eliminated *via* the urinary tract (2). Deficiency of this vitamin (ariboflavinosis) can provoke damage to the liver or skin, as well as cerebral changes, and alteration of glucose metabolism. Riboflavin is also involved in the prevention of migraine, anemia, cancer, hyperglycemia, hypertension, diabetes mellitus, and directly or indirectly in oxidative stress (2, 3).

The recommended daily dose of riboflavin for a healthy adult is in the range 0.9–1.6 mg (4, 5). In developed countries these dosage levels can be achieved *via* a balanced diet, as vitamin B_2_ is present in green vegetables, cereals and dried fruits, and also from eggs, meat and dairy products (6). Nevertheless, ariboflavinosis is a problem in underdeveloped countries, and riboflavin supplements are required by population groups at high risk of deficiency due to specific diets, like vegans/vegetarians (low intake or complete exclusion of dairy products and meat) or pregnant women (especially those with lactose intolerance and/or little meat intake) (7). Furthermore, processing and cooking of vegetable products causes a loss of B-group vitamins, including riboflavin (8).

Some food grade lactic acid bacteria (LAB) synthesize vitamin B_2_. Thus, fermentation with these LAB offers opportunities to improve the nutritional value of food products and the development of novel foods with an enhanced vitamin content. In addition, the adaptability of LAB to fermentation processes, their biosynthetic capacity and metabolic versatility are features that advocate their industrial application for producing and/or increasing riboflavin concentration in foods.

In order to isolate riboflavin-overproducing strains, the toxic compound roseoflavin (a structural analog of riboflavin) has been widely used as a selection agent for isolating spontaneous riboflavin-overproducing mutants, mainly belonging to *Lactococcus lactis*, *Lactiplantibacillus plantarum* (previously *Lactobacillus plantarum*), *Limosilactobacillus fermentum* (previously *Lactobacillus fermentum*), and *Leuconostoc mesenteroides* species (9–12). Fermented products made with the riboflavin-overproducing strains have been reported as a convenient and efficient food-grade biotechnological procedure (11, 13–18).

Some LAB are also able to produce exopolysaccharides (EPS), including dextrans, which have many industrial applications (19–25). The high molecular weight dextran produced by LAB (e.g., *L. mesenteroides* and lactobacilli), as well as by *Saccharomyces cerevisiae*, were labeled as “food grade” by EFSA in the year 2001 (21). These biopolymers possess a linear backbone composed of glucopyranosides with α-(1→6) linkages in the principal chain and with variable percentages of α-(1→4), α-(1→3), or α-(1→2) branches (20). Dextrans are hydrocolloid-like compounds that retain water and increase the viscosity of a food matrix without affecting taste (22). Therefore, dextrans are widely used as food additives (23). For example, they are used to increase palatability: (i) by the bakery industry and (ii) in the production of ice-creams, milk shakes, etc., (24). Various authors have demonstrated the improvement of bread quality by using dough enriched with dextrans (25, 26). LAB dextrans can affect the technological properties of doughs and breads by improving moisture retention and rheology, and increasing the final volume, the softness of the crumb and the shelf life of the final product (22). Furthermore, the addition of enzymatically produced dextran improved the volume and the texture of white bread, and also of bread containing 20% of rye flour (27). Due to their ability to bind water and to retain the CO_2_ produced during dough fermentation, dextrans can mimic the viscoelastic properties of gluten, making these bacterial EPS highly suitable for the manufacture of gluten-free or low-gluten bakery products (22). A dextran-rich sourdough obtained using a specific *L. mesenteroides* strain gave rise to several kinds of baked goods (from wheat rich dough products to rye sourdough bread), which had improved freshness, crumb structure, mouthfeel, and softness (28). Dextrans produced *in situ* by three LAB strains (belonging to *W. cibaria*, *W. confusa*, and *L. fermentum* species) isolated from sorghum significantly improved the rheological properties of a dough made of sorghum and wheat flour (29). Also, the addition of 20% sourdough fermented with a dextran-producing *W. cibaria* strain reduced significantly crumb hardness in breads based on buckwheat, teff, quinoa and wheat flours, as well as the staling rate in buckwheat, teff and wheat sourdough breads (30). High molecular weight dextrans synthesized by LAB are immuno-stimulants *in vitro* and appear to have anti-inflammatory properties, supporting their ability to improve the functionality of various products, including the preparation of fermented functional foods (31).

For the above reasons, the food industry is increasingly interested in obtaining LAB strains for the *in situ* production of dextran during food processing. If these strains were also able to produce riboflavin, then the industrial interest would be even higher. With this aim, we have previously isolated from mother doughs made from rye, three strains of *W*. *cibaria* (BAL3C-5, BAL3C-7 y BAL3C-22), which produce riboflavin and high levels of dextran (32). In this work, these three strains were used for selection of three riboflavin-overproducing *W. cibaria* strains (named BAL3C-5 B2, BAL3C-7 B2, and BAL3C-22 B2). The ability of these B2 strains to synthesize high levels of riboflavin and dextran has been tested and validated both under laboratory growth conditions and with the manufacture of experimental breads.

## Materials and methods

### Bacteria and growth conditions

The *W. cibaria* strains used in this work, and their characteristics, are detailed in Table 1. The bacteria were grown at 30°C without shaking in MRS medium (Man, Rogosa, and Sharpe medium, Condalab, Spain), MRS supplemented with 5% sucrose (MRSS), BD Difco™ Riboflavin assay medium (RAM, Thermo Fisher Scientific, USA) containing 2% glucose or RAM supplemented with 2% sucrose (RAMS). The LAB strains were grown in test tubes in a water bath or in microtiter plates (Sterile 96-Well Optical White w/Lid Cell Culture, Thermo Fisher Scientific, Rochester, NY, United States) in a Varioskan Flask System (Thermo Fisher Scientific, Waltham, MA, United States). The bacterial growth was determined by measurement of the optical density at 600 nm (OD_600 nm_). The growth rate (μ) of the LAB in liquid media was determined as previously described (32). The LAB CFU/mL of the liquid cultures was determined by plating 100 μL aliquots of the appropriate dilutions in MRS-agar medium and further incubation at 30°C for 48 h.

**TABLE 1:** Bacterial strains used in this work.

*W. cibaria* strains	Characteristics	Source of isolation	FMN riboswitch	Reference
BAL3C-5	Riboflavin-and dextran-producer	Fermented rye dough	Wild-type	(33)
BAL3C-7	Riboflavin-and dextra-producer	Fermented rye dough	Wild-type	(33)
BAL3C-22	Riboflavin-and dextran-producer	Fermented rye dough	Wild-type	(33)
BAL3C-5 B2	Riboflavin-overproducer and dextran-producer	Spontaneous mutant of BAL3C-5 selected by roseoflavin treatment	G35T mutant	This work
BAL3C-7 B2	Riboflavin-overproducer and dextran-producer	Spontaneous mutant of BAL3C-7 selected by roseoflavin treatment	G129A mutant	This work
BAL3C-22 B2	Riboflavin-overproducer and dextran-producer	Spontaneous mutant of BAL3C-22 selected by roseoflavin treatment	C43T mutant	This work

### Selection of riboflavin-overproducing strains

The *W. cibaria* BAL3C-5, BAL3C-7, and BAL3C-22 strains were individually grown in MRS medium to an OD_600 nm_ of 1.5. Then, the bacterial cultures were diluted 1:100 in RAM medium supplemented with roseoflavin (10 μg/mL) and grown until the end of the exponential phase. Afterward, the LAB were exposed to increasing concentrations of roseoflavin (50, 75, 100, 150, and 200 μg/mL) by sequential dilution and further growth in RAM supplemented with the riboflavin homolog. The BAL3C-7 and BAL3C-22 cultures grew even in the presence of roseoflavin at 200 μg/mL, whereas *W. cibaria* BAL3C-5 showed tolerance up to 150 μg/mL of the toxic compound. The roseoflavin resistant cultures were plated on MRS-agar and incubated for 48 h. Subsequently, three colonies from each roseoflavin-treated parental strain were randomly chosen to evaluate their riboflavin production, and the highest producer of each group was selected and designated as BAL3C-5 B2, BAL3C-7 B2, and BAL3C-22 B2, respectively. Finally, the bacteria present in the selected colonies were recovered by growth in liquid MRS and stored at −80°C in MRS supplemented with glycerol at 20%.

### Determination and analysis of the deoxyribonucleic acid sequence of the *rib* operons and of the *dsr* genes

The determination of the DNA sequences from the LAB genomes was performed by the “chain termination method” (Sanger sequencing), with a strategy of “primer walking” and utilizing PCR fragments containing either the *rib* operons or the *dsr* genes as DNA template. The oligonucleotides used are reported in Supplementary Table 1. The assembled and annotated DNA sequence reads have been deposited in the NCBI-GenBank. The GenBank accession numbers of the *dsr* genes are: ON677429 (BAL3C-5), ON677430 (BAL3C-7), ON677431 (BAL3C-22), ON677432 (BAL3C-5 B2), ON677433 (BAL3C-7 B2), and ON677434 (BAL3C-22 B2). The GenBank accession numbers of the *rib* operons are: ON420949 (BAL3C-5), ON420950 (BAL3C-7), ON420951 (BAL3C-22), ON420953 (BAL3C-5 B2), ON420954 (BAL3C-7 B2), and ON420952 (BAL3C-22 B2). The obtained DNA sequences of the *dsr* genes and the *rib* operons were compared with those of the selected *W. cibaria* and *W. confusa* strains held in GenBank using the BLASTN software (33). Sequences were aligned using the ClustalW software (34). Phylogenetic trees were made/inferred using the neighbour joining analysis (35). Evolutionary analysis was conducted using MEGA11 software (36).

Gene analysis was performed with the EditSeq ^®^ and SeqBuilder Pro ^®^ software (Version 15.3, DNASTAR, Madison, WI, USA) to infer the amino acid sequences of their products. The sequence reads were assembled and compared by using the SeqMan Pro ^®^ and MegAlign ^®^ software (Version 15.3, DNASTAR, Madison, WI, USA), respectively.

Secondary structure predictions of the sensor domain of the FMN riboswitch were obtained by using the RNAfold web server (The ViennaRNA Web Services, version 2.4.18). RNA secondary structure drawings were performed with VARNA 3.9 software (37).

The Signal P 6.0 server was used to predict the leader peptide of the Dsr and the location of the processing site in the *W. cibaria* strains (38).

### *In situ* detection of dextransucrases (Dsr) produced by the BAL3C-5 B2, BAL3C-7 B2, and BAL3C-22 B2 strains by zymogram

The riboflavin-overproducing LAB were grown overnight in MRS. Then, after sedimentation by centrifugation at 9,000 × *g* for 10 min, the bacteria were used to inoculate either RAMS or RAM to give an initial OD_600 nm_ of 0.1. The cultures were further incubated at 30°C for 24 h. Afterward, the bacteria were sedimented by centrifugation and the supernatants were subjected to PAGE in an 8% SDS-polyacrylamide gel at constant voltage (100 V). Afterward, Dsr activity was assayed *in situ* following the method of Miller and Robyt (39) with the modifications previously described (40). Briefly, the method used included a washing step for SDS removal, synthesis of the dextran by Dsr in sodium acetate buffer supplemented with 10% sucrose during 17 h and detection of the Dsr activity by staining with periodic acid-Schiff staining. To estimate the molecular weight of Dsr, the pre-stained Precision Plus Protein Dual Color Standards (Bio-Rad, CA, USA) including 15 polypeptides in the range of 10–250 kDa was used.

### Analysis of riboflavin and dextran produced by bacterial cultures grown in RAMS and RAM medium

The cells from overnight cultures of *W. cibaria* strains grown in MRS were sedimented by centrifugation at 9,000 × *g* for 10 min and used to inoculate either RAMS or RAM to give an initial OD_600 nm_ of 0.1 and the bacteria were further grown at 30°C as indicated in the Results section.

The fluorescence of the riboflavin present in the bacterial cultures or supernatants was measured upon excitation at a wavelength of 440 nm and detection of emission at a wavelength of 520 nm by using a Varioskan Flask System (Thermo Fisher Scientific, USA). Finally the concentration of the riboflavin was determined using a calibration curve as previously described (12).

The dextran present in the culture supernatants was precipitated with three volumes of absolute ethanol and washed twice with 80% (v/v) ethanol and its concentration estimated as neutral carbohydrate content determined by the phenol–sulphuric acid method using a glucose calibration curve (41).

### Laboratory production of experimental breads

The LAB were grown in MRSS at 30°C for 2–3 h with low and constant aeration. After sedimentation of the bacterial cells by centrifugation and two washings with 0.9% saline solution, the cultures were diluted prior to inoculation of the dough. To generate the doughs, refined organic wheat flour with strength W200, from Molinos del Duero y Compañía General de Harinas, S.L. (Carr. de Villalpando, 13, 49029 Zamora) was used. The doughs contained 400 g of flour, 300 mL of water, 5% sucrose, and 0.64% NaCl, and were prepared in a dough mixer. Subsequently, the doughs were divided into portions of 50 g and inoculated with the corresponding LAB [1 × 10^9^ colony forming units (CFU)/mL]. After kneading, each inoculated dough portion was subdivided in another three portions of 15 g each, then fermentation was carried out at 30°C for 16 h and the experimental breads were generated in triplicate by baking at 210°C for 15 min. Prior to baking, the final concentration of LAB (CFU/g) in the fermented dough was determined by plating (Supplementary Table 2).

### Analysis of riboflavin, flavins, and dextran concentrations in the laboratory breads

The laboratory prepared breads would contain free riboflavin and other flavins synthesized by the LAB as well as flavins naturally present in the flours. Therefore, soluble riboflavin was extracted and quantified, as well as any other flavins converted into riboflavin prior to quantification. Also, dextran produced by the LAB could be in a soluble or insoluble form. Therefore, soluble and total dextrans were independently extracted and quantified.

To extract free riboflavin and soluble dextran from the breads, samples (1.5 g) were placed in 15 mL Falcon™ (Corning Science, México) tubes and distilled water (3 mL) was added. After vigorous vortexing, samples were incubated at 20°C for 24 h. Then, to convert dextran into isomaltose 150 μL of a solution containing 0.18 g of *Chaetomium erraticum* dextranase (Sigma-Aldrich, Darmstadt, Germany) was added and samples were incubated at 30°C for 18 h, centrifuged at 8,000 × *g* for 10 min, and supernatants filtered by using a 0.22 μm filter. Afterward, aliquots were stored at −20°C until further analysis.

To extract flavins from the breads and to convert them into riboflavin, samples (1 g) were placed in 25 mL flasks, and 0.1 M HCl (10 mL) was added. Then, the samples were autoclaved at 121°C for 30 min. Subsequently, the pH of the suspensions was neutralized to a pH 6.5 by addition of 1.4 mL of 4 M sodium acetate pH 9.5. Furthermore, to convert dextran into isomaltose, 0.5 mL of a solution containing 0.6 g of *C. erraticum* dextranase was added and the samples were incubated at 30°C for 18 h. To remove solid residues, the samples were centrifuged at 8,000 × *g* for 10 min, and the supernatants filtered through a cheesecloth and subsequently through a 0.22 μm filter. Afterward, the filtrate was aliquoted and kept frozen at −20°C until further analysis.

The concentration of riboflavin in the processed samples was determined by measuring its fluorescence as described in section “Analysis of riboflavin and dextran produced by bacterial cultures grown in RAMS and RAM medium”, as well as by chromatographic analyses. These latter analyses were performed with a HPLC equipment composed of a degasser system, a quaternary pump, an automated injector, a column oven, an ultraviolet–visible diode array detector (UV–vis-DAD) and a fluorescence detector (FLD) (Agilent-1200/1260 Infinity II Series, Palo Alto, CA, USA). A Kinetex EVO C 18 100 Å 4.6 × 150 mm, 5 μm internal diameter analytical column with a SecurityGuard ULTRA Cartridges UHPLC C18 (Phenomenex, Torrance, CA, USA) thermostated at 40°C, was used for the analytical determination of riboflavin. A ChemStation computer software (Agilent, Palo Alto, CA, USA) recorded signals. HPLC analyses were achieved by an isocratic elution at 0.6 mL/min using the conditions described by Jakobsen et al. (42), with a mobile phase constituted by a methanol/water (40:60 v/v) mixture, freshly prepared every day. A fluorescence detector set at an excitation wavelength of 449 nm and an emission wavelength of 516 nm monitored the eluate. Spectral analyses of the riboflavin standard and samples were performed to verify the method’s selectivity.

The total dextran concentration was determined by quantification of the isomaltose generated by the polymer hydrolysis by gas chromatography-mass spectrometry (GC-MS) using myo-inositol as internal standard, after derivatization with hydroxylamine chloride in pyridine to form the oxime of the isomaltose and generation of the trimethylsilylated derivative by treatment with bis-trimethylsilyl trifluoroacetamide as described (40). Quantification of isomaltose concentration was performed according to peak area, corrected with the response factors calculated for each compound using the internal standard and the software GC-ChemStation Rev. E.02.00 (2008) from Agilent (Palo Alto, CA, USA).

### Statistical analysis

Exopolysaccharides (EPS) produced by the strains in growth media was quantified using the phenol-sulphuric acid method. *T*-tests were performed to determine if the values of the parental and the mutant strains were significantly different, and *p*-values were adjusted for multiple testing by the Benjamini and Hochberg method (43). In addition, differences between groups for EPS production by all strains (mutant and parental), as well as for total and soluble dextran fractions in breads, were performed with a one-way analysis of variance, and mean pairwise comparisons were computed with a Tukey’s test. Results are marked with letters and means with the same letter are not significantly different (α = 0.05). All analyses were performed with the R software version 4.1.3 (44).

In the case of evaluation of riboflavin production in growth medium, for every parental-mutant pair, the effects of strains, culture media, and their interaction were analyzed with a two-way analysis of variance. A *p* value ≤ 0.05 was considered significant. When interactions were significant, independent *t*-tests were performed for each medium, and *p*-values were adjusted for multiple testing by the Benjamini and Hochberg method (43). Again, to establish differences between groups for the riboflavin produced by all strains (mutant and parental) as well as for flavins and free riboflavin in breads, a one-way analysis of variance was performed and mean pairwise comparisons were computed with a Tukey’s test. Results are shown with letters and means with the same letter are not significantly different (α = 0.05). All analyses were performed with the R software version 4.1.3 (44).

## Results and discussion

### Selection and analysis of *W. cibaria* riboflavin-overproducing strains

Three *W. cibaria* strains (BAL3C-5, BAL3C-7, and BAL3C-22) previously isolated from fermented rye dough and characterized for their ability to produce both dextran and riboflavin (32) were selected in this study and treated with roseoflavin, with the aim to identify riboflavin-overproducing strains, potentially useful for the production of functional bread enriched in riboflavin and dextran.

The three parental strains were independently subjected to cycles of treatment with increasing concentrations of roseoflavin and three strains resistant to the riboflavin homologue (one from each wild-type treated strain) were obtained. These spontaneous mutants were designated as BAL3C-5 B2, BAL3C-7 B2, and BAL3C-22 B2 and, using their parental strains as control, were analyzed to determine their capability to produce riboflavin and dextran in liquid medium. The RAMS medium (containing 2% sucrose and lacking riboflavin and polysaccharides), which we have shown to be suitable for the analysis of the riboflavin and dextran produced by BAL3C-5, BAL3C-7, and BAL3C-22 strains (33), was used.

The Dsr from LAB produce extracellularly dextran by hydrolysis of sucrose molecules coupled to the transfer of glucose to the nascent α-glucan polymer (16). Moreover, we have shown that riboflavin produced by *W. cibaria* BAL3C-5, BAL3C-7, and BAL3C-22, as is the case in other LAB (12, 45), can be detected and quantified in culture supernatants (32). Therefore, after growing the six LAB for 23 h in RAMS, the content of dextran and riboflavin present in the culture supernatants was determined, and the final biomass estimated by measuring the OD_600 nm_ and by plating (Table 2).

**TABLE 2:** Comparative analysis of riboflavin and dextran production by the wild-type and mutant *W. cibaria* strains in RAMS and RAM media.

Medium	RAMS		RAMS		RAM		RAMS	RAM	RAMS	RAM

Compound strain	^1^Dextran		^2^Riboflavin				OD_600 nm_		CFU/mL	
			
	Concentration (mg/mL)	^3^B2/wt ratio	Concentration (μ g/mL)	^3^B2/wt ratio	Concentration (μ g/mL)	^3^B2/wt ratio				
BAL3C-5 B2	5.61 ± 0.21^B^	0.88	3.45 ± 0.04^a^	19.17	1.82 ± 0.01^α^	60.67	3.2	1.9	1.3 × 10^9^	7.2 × 10^8^
BAL3C-5	6.35 ± 0.22^A^		0.18 ± 0.01^d^		0.03 ± 0.01^△^		2.8	1.6	1.2 × 10^9^	6.5 × 10^8^
BAL3C-7 B2	5.74 ± 0.20^B^	0.88	2.53 ± 0.06^b^	14.05	1.35 ± 0.01^β^	45.00	3.0	2.0	1.9 × 10^9^	9.2 × 10^8^
BAL3C-7	6.55 ± 0.21^A^		0.18 ± 0.01^d^		0.03 ± 0.01^△^		2.8	1.6	1.4 × 10^9^	6.8 × 10^8^
BAL3C-22 B2	6.66 ± 0.22^A^	1.01	1.66 ± 0.08*^c^*	8.74	0.84 ± 0.02^γ^	28.00	3.0	2.1	1.7 × 10^9^	8.4 × 10^8^
BAL3C-22	6.61 ± 0.17^A^		0.19 ± 0.01^d^		0.03 ± 0.00^△^		2.8	1.6	1.4 × 10^9^	5.6 × 10^8^

^1^Dextran concentration in culture supernatants was determined by measuring neutral sugars concentration after ethanol precipitation. The values are expressed as mean ± standard deviation (SD) of three independent experiments.

^2^Riboflavin concentration present in the culture supernatants was inferred by measuring its fluorescence and with a riboflavin calibration curve. The values are expressed as mean ± standard deviation (SD) of three independent experiments.

^3^*W. cibaria* B_2_ mutant/wild-type ratio for riboflavin concentration determined in the culture supernatants. Values with the same superscript letter have no statistically significant divergences (p value ≤ 0.05).

The six strains produced similar levels of dextran, ranging from 5.61 to 6.66 mg/mL with BAL3C-22 B2 being the highest producer. Moreover, statistical analysis revealed that only BAL3C-5 B2 and BAL3C-7 B2 produced slightly lower levels of dextran compared to the parental BAL3C-5 (*p* = 0.0323) and BAL3C-7 (*p* = 0.0209) (Table 2 and Supplementary Figure 1).

With regards to the riboflavin levels, bacterial cultures of the six strains grown in RAMS or RAM were tested. The B2 strains produced statistically significant higher levels of riboflavin than their corresponding or the other two parental strains (*p* = 7.33 × 10^–20^ or *p* = 3.45 × 10^–21^ in RAMS or RAM) (Table 2 and Supplementary Figures 2, 3). After 23 h of growth in RAMS (containing glucose plus sucrose) *versus* in RAM (containing only glucose), all the strains reached a higher OD_600 nm_ (3.2–2.8 in RAMS and 2.1–1.6 in RAM) correlating with higher levels of CFU/mL (1.9 × 10^9^–1.2 × 10^9^ in RAMS and 9.2 × 10^8^–5.6 × 10^8^ in RAM) and consequently produced higher levels of riboflavin. Apart from that, the behavior of the strains was the same in both media, with the highest levels of riboflavin being synthesized and released to the media by BAL3C-5 B2 (3.45 and 1.82 μg/mL in RAMS and RAM, respectively). In addition, the increase of the riboflavin production by the B2 mutants compared with the production of the corresponding parental strains was more pronounced in RAM (ranging from 28- to 61-fold) than in RAMS (ranging from 8- to 19-fold), with the lowest being for the pair BAL3C-22 B2 and BAL3C-22.

**FIGURE 1:**
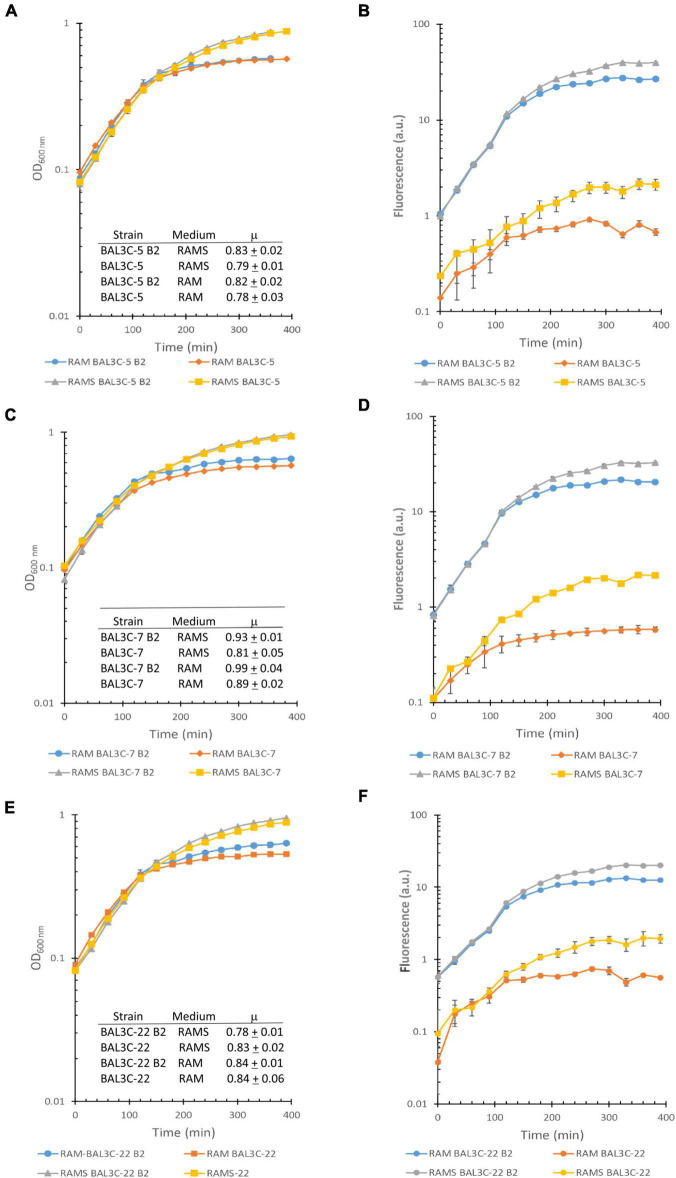
Detection of riboflavin production by the *Weissella cibaria* strains. Real time monitoring of growth **(A,C,E)** and riboflavin **(B,D,F)** production of the different *W. cibaria* strains analyzed in this study [BAL3C-5 and BAL3C-5 B2 **(A,B)**; BAL3C-7 and BAL3C-7 B2 **(C,D)**; BAL3C-22 and BAL3C-22 B2 **(E,F)** in RAM or in RAMS]. In the left panels, the plots display the growth curves of each strain, with the symbols and error bars representing, respectively, the average and standard deviation of three independent experiments. Growth rate constants (μ) were calculated during the exponential phase for each of the strains in the two-culture medium. The μ values are expressed as mean ± standard deviation of three independent experiments. In the right panels, the plots display the riboflavin production curves of each strain. Symbols and error bars represent, respectively, the average and standard deviation of three independent riboflavin fluorescence measurements.

Furthermore, a simultaneous analysis of the bacterial growth in RAM or in RAMS by measuring their OD_600 nm_ and production of riboflavin by fluorescence detection in real time was performed and the growth rates during the exponential phase of growth inferred (Figure 1). The highest μ was observed for the BAL3C-7 B2 strain when grown in either RAM (μ = 0.99 h^−1^) or RAMS (μ = 0.93 h^−1^) medium and the lowest for BAL3C-5 in both RAM (μ = 0.78 h^−1^) and RAMS (μ = 0.79 h^−1^). Moreover, correlating with the data presented in Table 2, the six strains reached a higher final biomass in RAMS than in RAM. In addition, as expected, the BAL3C-5 B2, BAL3C-7 B2, and BAL3C-22 B2 strains produced high levels of riboflavin in either RAMS or RAM media and during both the exponential and stationary phases of growth.

Taken together our results reveal that the *W. cibaria* BAL3C-5 B2, BAL3C-7 B2, and BAL3C-22 B2 strains overproduce riboflavin and maintain the capability to produce dextran.

### Characterization of the mutations of the riboflavin-overproducing strains

**FIGURE 2:**
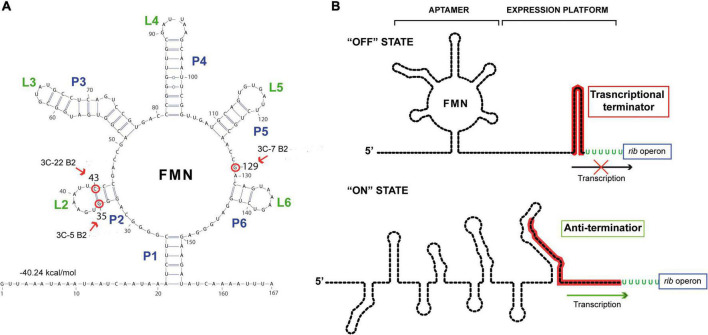
Secondary structure predictions of the riboswitch domains. **(A)** Secondary structure prediction obtained by using RNAfold web server with the sequence of the nascent *rib* operon mRNA of *Weissella cibaria* BAL3C-5. The free energy associated with this aptameric conformation is shown. Red arrows and red circles indicate altered ribonucleotides in the aptamer in BAL3C-22 B2 (U at position 43), BAL3C-5 B2 (U at position 35) and BAL3C-7 B2 (A at position 129) due to the mutations present in the DNA of these strains. The strain containing the base change is indicated in each case. **(B)** Schematic representation of the possible “ON” and “OFF” conformations of the FMN riboswitch. The FMN riboswitch mechanism of gene expression is based on ligand binding and signal transduction through conformational changes from the “ON” state (anti-terminator structure) to the “OFF” state (transcriptional terminator). Aptamer and expression platform domains are indicated over the “OFF” state structure. The sequence involved in the formation of the transcriptional terminator is red colored. Arrows indicate transcription of the *rib* operon.

The enzymes involved in the riboflavin biosynthetic pathway are encoded by the *rib* operon composed of the *ribG*, *ribB*, *ribA*, and *ribH* genes, whose expression is regulated by a transcriptional FMN riboswitch located in the 5’ untranslated region of the messenger RNA. This regulatory element consists of a sensor domain (the aptamer) that contains five hairpins (P2/L2 to P6/L6) and is closed by the P1 basal helix (Figure 2A), whose 3’-end is connected to the regulatory domain (or expression platform). The regulatory domain is predicted to adopt two alternative configurations that correspond to the ON state (characterized by an anti-terminator secondary structure) and to the OFF state, which includes an intrinsic transcriptional terminator preventing expression of the *rib* operon [Figure 2B; (45)]. Binding of the effector (FMN) to the aptamer induces a conformational change in the expression platform from the ON to the OFF state, thus avoiding the metabolic burden of expressing the genes involved in riboflavin biosynthesis when not required.

Roseoflavin-resistant mutants of LAB usually harbor mutations that impair the regulatory activity of the *rib* operon riboswitch, enabling the bacterial cells to synthesize riboflavin in the presence of either FMN or roseoflavin. Therefore, to localize and characterize the mutations of the riboflavin-overproducing *W. cibaria* strains, the DNA sequence of the entire *rib* operon, including the FMN riboswitch, was determined for each of them as well as for the respective parental strains. No differences were detected between the DNA sequences of the *rib* operons of the three parental strains. The operons of BAL3C-5 B2, BAL3C-7 B2, and BAL3C-22 B2 each showed only one mutation located in the untranslated region of the *rib* mRNA.

Analysis of the folding of this region with the RNAfold software predicted the existence of an FMN riboswitch aptamer (matching the consensus sequence and structure) where the three mutations were located (Figure 2A). In this context, strain BAL3C-5 B2, which showed the highest production of riboflavin in liquid growth media, carries in its DNA the mutation G35T, and as a consequence in its FMN riboswitch there is a U instead of a G at the position 35 (position 1 corresponding to the putative start site of the *rib* mRNA), a change that would destabilize the P2 stem of the aptamer. The BAL3C-22 B2 strain has the lower riboflavin-overproducing phenotype and contains the mutation C43T, that results in an aptamer with a U43 instead of the C43 (which is predicted to pair with the G35 in the wild-type riboswitch). This mutation should also affect the stability of the P2 helix, although to a lesser extent, because a base pair GU could still be formed. Finally, BAL3C-7 B2 carries the mutation G129A, that results in the change of one ribonucleotide in the aptamer at position 129. Previous 3D crystallographic studies performed with the FMN riboswitch of *Fusobacterium nucleatum* have demonstrated that the same relative position in the aptamer is involved in the direct interaction with the FMN effector (46).

The location of the mutations in BAL3C-5 B2, BAL3C-7 B2, and BAL3C-22 B2 indicates that these changes could impair or prevent the binding of FMN to the aptamer, thereby avoiding its inhibitory effect and being responsible for the riboflavin-overproducing phenotype of these strains.

To support this hypothesis, growth, and production of riboflavin by the mutant and parental strains in RAMS supplemented with FMN was analyzed in real time (Supplementary Figure 4). All the strains showed the same growth pattern (Supplementary Figure 4A). The addition of FMN altered the pattern of fluorescence of the cultures of the three parental strains ascribed to flavins production during growth. By contrast, with the results obtained in absence of FMN (Supplementary Figure 4B), with detection of riboflavin production from the beginning of the growth (Figures 1B,D,F), the fluorescence decreased during the early stage of growth, as we have previously detected for *L. plantarum* wild–type strains (12), and did not start to increase until the middle of the exponential phase (Supplementary Figure 4), as we have previously observed for these *W. cibaria* strains in the presence of riboflavin (32). These results were expected due to an inhibitory effect of the FMN, upon internalization, in the riboflavin biosynthesis, proceeding to a latter expression of the *rib* operon when levels of the effector are exhausted in the cells. However, as expected the presence of the FMN had no influence on riboflavin production by the three mutant strains, since increase of fluorescence due to the presence of flavins was observed from the beginning of the exponential growth phase, as we have previously observed for riboflavin-overproducing *L. plantarum* mutants also carrying point mutations located in its corresponding aptamer of the FMN riboswitch (45). Consequently, these results and the fact that no mutations were found in the *rib* operon encoding the enzymes involved in the riboflavin biosynthetic pathway, strongly suggest that the mutations in the FMN-riboswitch are responsible for the riboflavin-overproducing phenotype of the *W. cibaria* B2 strains. However, to prove this hypothesis a deep transcriptional analysis has to be performed in a future work, as has been already done for a *L. plantarum* riboflavin-overproducing mutant (45).

In addition, we performed a comparative analysis of the *rib* operon sequences of some *W. cibaria* and *W. confusa* strains whose genome sequences are publicly available. In this analysis, the *rib* operons of BAL3C-5, BAL3C-7, and BAL3C-22 were included. The DNA sequence of the *rib* operon from the three *W. cibaria* parental strains showed 100% identity with that of *W. cibaria* CH2, a strain isolated from cheese of the occidental Himalayas. Usually, phylogenetic investigations of the genus *Weissella* are based on *rrs* (encoding the 16S rRNA) and/or *pheS* genes sequences (32, 47). Recently, a comparative genomic analysis of *Weissella* species allowed the construction of a whole genome phylogenetic tree based on single-copy core orthologs (48). Either based on single gene sequences or whole genome comparisons, *W. cibaria* and *W. confusa* appear to be phylogenetically closely related, grouping together in the same cluster.

**FIGURE 3:**
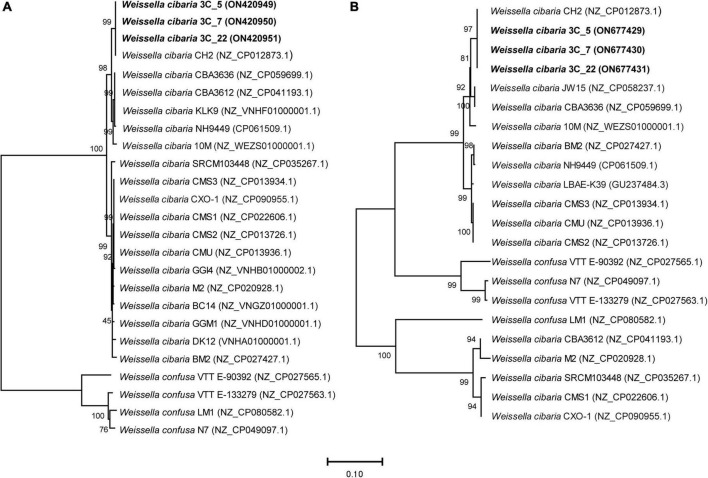
Neighbor-joining phylogenetic rooted trees based on the sequences of the *rib* operon genes [3,290 nt; panel **(A)**] and of the *dsr* genes [4,546 nt; panel **(B)**], showing the taxonomic location of the analyzed strains. The percentage of replicate trees in which the associated taxa clustered together in the bootstrap test (1,000 replicates) are shown above the branches (64). The tree is drawn to scale, with branch lengths in the same units as those of the evolutionary distances used to infer the phylogenetic tree. The evolutionary distances were computed using the Maximum Composite Likelihood method (65) and are in the units of the number of base substitutions per site. This analysis involved 25 nucleotide sequences in the case of the *rib* operon sequences and 22 for the *dsr* gene sequences. All ambiguous positions were removed for each sequence pair (pairwise deletion option). The total number of positions in the final dataset was 3,650 and 4,584 position for the *rib* operon and *dsr* analysis, respectively. Accession numbers from GenBank are given in brackets.

The phylogenetic relationship of BAL3C-5, BAL3C-7, and BAL3C-22 with other *W. cibaria* and *W. confusa* strains based on *rib* operon sequences is depicted in Figure 3A. In spite of the high degree of *rib* operon sequence similarity shared by all the strains, BAL3C-5, BAL3C-7, and BAL3C-22 grouped together with CH2 in a separate branch, as expected by its 100% sequence identity. It is worth noting that all *W. cibaria* and *W. confusa* strains included in the analysis were placed in divergent branches although, as stated before, the two species belong to the same phylogenetic cluster. This fact may support the use of the *rib* operon as marker for the discrimination between *Weissella* species in future phylogenetic studies.

### Identification of the *dsr* genes and detection of active Dsr enzymes synthesized by the mutant strains

Only one Dsr enzyme encoded by a *dsr* gene is required for the synthesis of the dextran. Therefore, the DNA sequences of the *dsr* genes from the three parental and the three mutant strains were determined. The genes of the six strains had a length of 4,342 bp and were identical, and also 99% identical to the corresponding gene of *W. cibaria* CH2. We also analyzed the phylogenetic relationship of these strains with other *W. cibaria* and *W. confusa* strains, based on the *dsr* gene sequences (Figure 3B). As observed in the tree based on the *rib* operon sequences, *W. cibaria* BAL3C-5, BAL3C-7, and BAL3C-22 *dsr* genes grouped together with that of the CH2 strain in the same branch, confirming the close relationship between the four strains. However, this group is the exception when comparing the trees based on the *dsr* gene and on the *rib* operon, since different evolutionary relationships were detected. The most important observation was that all the *W. cibaria* strains analyzed were not placed together in the same divergent branch, indicating that the use of this marker will not discriminate between *W. cibaria* and *W. confusa* within the same phylogenetic cluster.

The inferred sequence of amino acids of Dsr showed that the *dsr* genes encode a protein of 159.099 kDa, which has an amino-terminal signal peptide involved in protein processing and secretion in Gram-positive bacteria. The analysis of the amino acid sequence of Dsr with the program Signal P 6.0 allowed us to predict that the processed extracellular Dsr has a molecular mass of 156.411 kDa.

**FIGURE 4:**
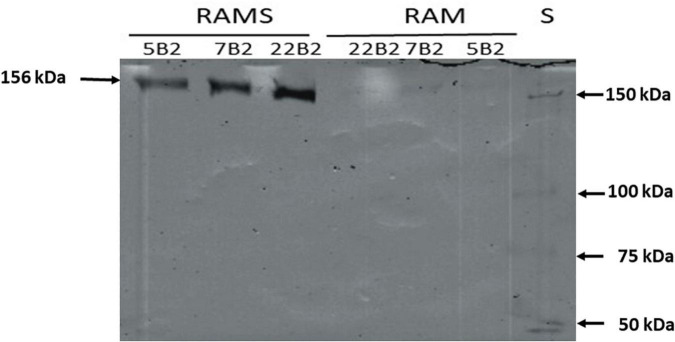
*In situ* detection of dextransucrase activity in cell free supernatants of LAB cultures. The indicated *Weissella cibaria* riboflavin-overproducing strains were grown in the presence of glucose [Riboflavin assay medium (RAM)] or glucose plus sucrose [Riboflavin assay medium with sucrose (RAMS)], the culture supernatants were subjected to SDS-PAGE and after protein renaturation, were analyzed *in situ* for Dsr activity. S, protein Mw standard.

Furthermore, since Dsr is extracellular, and with the aim of detecting the active form of the enzyme, supernatants of cultures of BAL3C-5 B2, BAL3C-7 B2, and BAL3C-22 B2 grown in RAMS or in RAM for 24 h were used to perform zymograms by *in situ* synthesis of dextrans after fractionation in SDS-polyacrylamide gel (Figure 4). The development of the gel revealed only one intense band at the expected position (156 kDa) in the culture supernatants of the three strains grown in RAMS and a very faint band in the samples grown in RAM. As a consequence, the results revealed that the production of active Dsr in this *W. cibaria* strains is induced when sucrose is present in the growth medium.

We have previously detected, with the same *in situ* methodology, this behavior for *W. cibaria* Av2ou, *W. confusa* FS54 and *Leuconostoc lactis* AV1n strains isolated from various Tunisian habitats (40). Moreover, in the case of *L. lactis* AV1n isolated from avocado, like for *L. mesenteroides* NRRL B-512F (49), it has been demonstrated that in the presence of sucrose induction of the *dsr* genes expression takes place at the transcriptional level (50). However, this is not a general feature of LAB, since zymogram analysis of soluble Dsr from five *W. cibaria* strains and one *W. confusa* isolated from sourdoughs revealed higher enzyme activity when the bacteria were previously grown in the presence of glucose instead of sucrose (51). This behavior was also observed for the *W. confusa* V30 strain isolated from an olive tree leaf (40) and for *Lactobacillus sakei* MN1 isolated from meat (52). Consequently, two different patterns of response to the presence of sucrose have been detected for the expression of the *dsr* of LAB independently of the isolation habitat including strains belonging to the *Weissella* genus and *W. cibaria* species.

### Evaluation of BAL3C-5 B2, BAL3C-7 B2, and BAL3C-22 B2 for experimental bread making

#### Preparation of experimental breads and extraction of dextran and flavins

**FIGURE 5:**
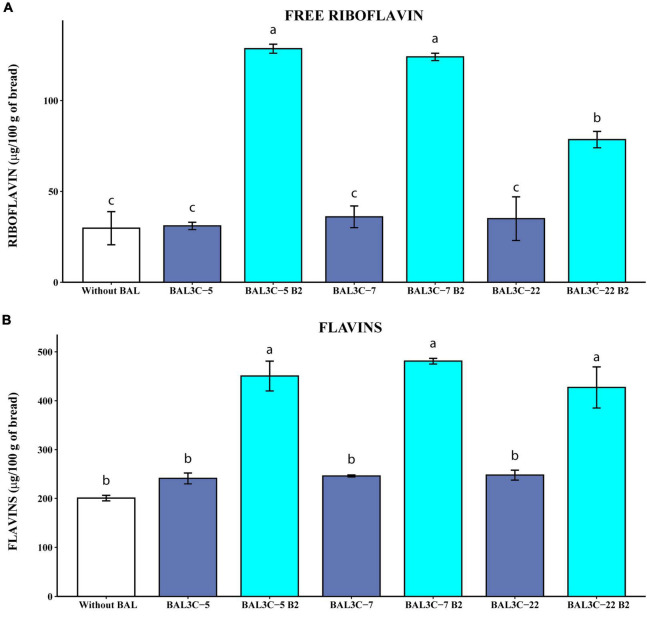
Determination of the free riboflavin and the flavin levels in breads produced with *Weissella cibaria* strains. Levels of water-soluble riboflavin (free riboflavin) **(A)** or riboflavin generated by acidic hydrolysis at high temperature of flavins (flavins) **(B)** present in the breads are depicted. The means of two determinations and the standard deviations are indicated. Values with different superscript letters indicate that the levels differed significantly (*p* ≤ 0.05, see details of statistical analysis in Supplementary Figure 6).

**FIGURE 6:**
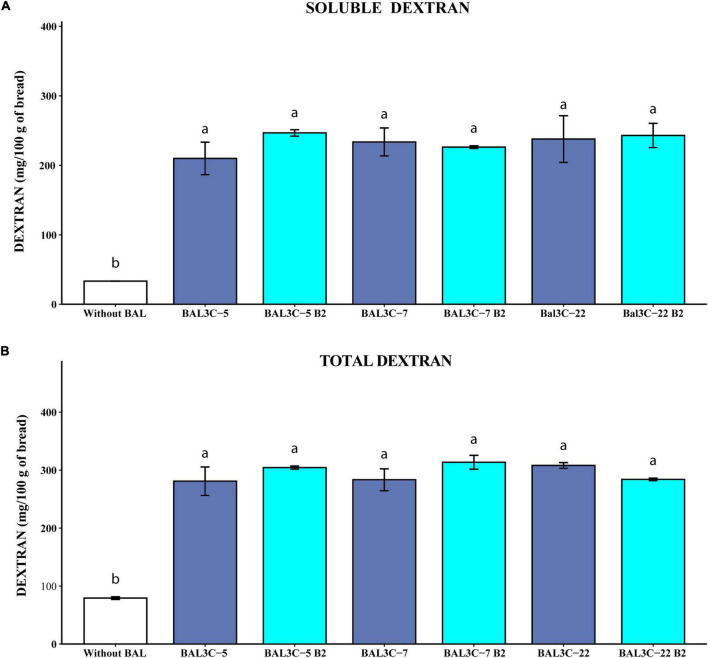
Determination of soluble and total dextran levels in breads produced with *Weissella cibaria* strains. Levels of water soluble dextran (soluble) **(A)** or dextran extracted after acidic and thermal treatments and hydrolyzed with dextranase (total) **(B)** present in the breads are indicated. The means of two determinations and the standard deviations are indicated. Different superscript letters indicate that the levels differed significantly (*p* ≤ 0.05, see details of statistical analysis in Supplementary Figure 7).

The riboflavin-overproducing and the parental *W. cibaria* strains were independently tested for their capability to produce riboflavin and dextran in wheat doughs supplemented with sucrose and fermented for 16 h. To induce the synthesis of the Dsr, the *W. cibaria* were grown in MRSS medium containing sucrose, prior to inoculation of the doughs. As a control, a dough without *W. cibaria* strains was used. After the fermentation period, LAB viability was evaluated by plating (Supplementary Table 2). In the control dough, a bacterial concentration of 4.37 × 10^6^ CFU/g was detected, whereas in all the other doughs inoculated with *W. cibaria* strains, the LAB concentration ranged from 9.1 × 10^8^–2.8 × 10^9^ CFU/g. Since the LAB were inoculated prior to fermentation, at a concentration of 1 × 10^8^ CFU/g, the results suggested a good survival and further growth of the inoculated *W. cibaria* strains during dough fermentation. Furthermore, after the baking process, breads were analyzed for their content in flavins and dextran (Figures 5, 6).

To determine specifically the concentration of the dextran without interference of other potential polyglucans present in bread, the *C. erraticum* dextranase was used to hydrolyze the polymer, and the isomaltose generated was detected and quantitated by GC-MS analysis (Figure 5 and Supplementary Figure 5). The dextranase of *C. erraticum* specifically hydrolyses dextran to isomaltose (53), and it has been previously used in a specific manner to convert dextran synthesized *in situ* by *W. confusa* in wheat sourdough into the disaccharide (54). However, in that particular case, the dextranase was used in conjunction with an α-glucosidase from *Aspergillus niger*, to convert the isomaltose in glucose, that was subsequently quantified (54).

In addition, two procedures were applied for the extraction of the compounds of interest. On one hand, free riboflavin and soluble dextran were extracted from the breads by incubation at 20°C for 24 h, upon water addition. Then, the riboflavin fluorescence (Figure 5A) and the levels of isomaltose generated by dextran hydrolysis (Figure 6A) were quantified. On the other hand, to determine levels of flavins and total dextran present in the breads, the extraction and conversion was performed at high temperature under acidic conditions and further treatment with the dextranase of *C. erraticum*. This procedure resulted in conversion of the flavins present in the sample into riboflavin (Figure 5B) and dextran to isomaltose (Figure 6B). Next, riboflavin was quantified by fluorescence spectrometry, directly (Figure 6) or after HPLC fractionation (Supplementary Table 3).

#### Free riboflavin and flavins content in experimental breads

The results obtained when the levels of riboflavin were measured without HPLC fractionation, revealed that the parental strains did not increase significantly the concentration of either free riboflavin or flavins (Figure 5) compared with those obtained by fermentation with only the dough microbiota. However, the riboflavin-overproducing strains increased with statistical significance the concentrations of free riboflavin (6.1- to 10-fold; *p* = 2.76 × 10^–5^) (Supplementary Figure 6A) and flavins (2.1- to 2.4-fold; *p* = 8.32 × 10^–5^) (Supplementary Figure 6B) over the control sample levels. Likewise, the three mutant strains enriched the breads in riboflavin and flavins more than their corresponding parental strains (Supplementary Figure 6). In addition, both BAL3C-5 B2 and BAL3C-7 B2 provided similarly high levels of free riboflavin (around 125 μg/100 g of bread). Also, in the breads obtained here by fermentation only with one of these two mutant LAB, in the absence of yeast, we detected around 465 μg of flavins converted into riboflavin/100 g, this value being similar to the 681 μg/100 g obtained with the riboflavin-overproducing mutant *L. plantarum* B2 of industrial interest during bread production in co-fermentation with yeast, taking into account that products obtained with yeast only contained up to 241 μg/100 g (55). Thus, a synergetic effect of *L. plantarum* B2 and yeast was observed, also detected for *L. fermentum* mutants (11) and for BAL3C-5 B2 and BAL3C-7 B2 strains (unpublished results). Furthermore, the levels of riboflavin in the breads produced with each of the 3 *W. cibaria* mutant strains (428–480 μg/100 g) were higher than that reported for Czech white bread (208 μg/100 g) (56) or for the top five white bread products high in riboflavin (337–383 μg/100 g).^1^ Thus, these results show a good ability of the riboflavin-overproducing *W. cibaria* to generate bread biofortified with vitamin B_2_.

We have previously shown that riboflavin produced by riboflavin-overproducing *L. plantarum* mutants in growth medium could be reliably quantified by direct measurement of its fluorescence without HPLC fractionation, and that the low levels produced by the parental strains could not be detected after the chromatographic fractionation (12). Therefore, to validate that the method used to convert flavins in riboflavin and the direct fluorescent detection of this compound were reliable, samples of the treated bread were first fractionated by HPLC and then the riboflavin was detected by fluorescence and quantified. Supplementary Table 3 depicts the levels of riboflavin detected by direct measurement of the fluorescence or after HPLC fractionation. After the chromatographic step, a fluorescent peak corresponding to riboflavin was observed in the samples of the breads fermented with the parental and mutant strains (result not shown), that was quantified. However, when the results obtained by the two methods were compared, we detected only similar levels for the breads fermented with BAL3C-5 B2, BAL3C-7 B2, or BAL3C-22 B2, the ratio of direct/HPLC levels being 0.81, 0.78, or 0.93, respectively (Supplementary Table 3). In the case of the breads produced with the parental strains BAL3C-5, BAL3C-7, and BAL3C-22, or spontaneously fermented without LAB inoculation, the ratios of the quantified riboflavin levels were 2.90, 1.5, 1.9, or 2.2, respectively. Therefore, these results validate the direct quantification method to determine concentration of flavins extracted from bread, and suggest that when the riboflavin production is low, it is more reliable than the HPLC method.

In recent years, riboflavin biofortification of fermented foods rather than vitamin supplementation has attracted great interest in the food industry, although as far as we know there are not yet any commercialized cereal products biofortified with vitamin B_2_ by LAB. In particular, *L. plantarum* and *L. fermentum* strains were successfully employed to obtain vitamin B_2_-enriched experimental bread (11, 14). Although in a previous study we reported on the selection of riboflavin-producing *W. cibaria* strains (32), this is the first work where robust vitamin-B_2_ overproducing derivatives belonging to this genera have been obtained. Indeed, apart from the widely reported applications of *L. plantarum* strains (57, 58), only few species of food-grade bacteria including *L. lactis* (9), *L. mesenteroides*, *P. freudenreichii* (10), *L. fermentum* (11) and *Limosilactobacillus reuteri* (59) have been reported for the vitamin B_2_ bio-enrichment of fermented foods endorsing the biotechnological importance of exploring the microbial biodiversity of LAB from different species and ecological niches.

#### Soluble and total dextran content in experimental breads

Supplementary Figure 5 depicts examples of representative chromatograms obtained from the GC-MS analysis of soluble dextran present in the wheat dough, and in breads obtained by fermentation with only the dough microbiota, or in presence of either BAL3C-22 B2 or BAL3C-22 (Supplementary Figures 5A,B). The peak corresponding to the isomaltose was detected in all breads analyzed (Supplementary Figures 5A–C), but not in the wheat dough (Supplementary Figure 5D). In addition, the dough contained maltose that was still present in all the breads. Moreover, production of lactic acid as well as high concentration of fructose (presumably generated by the catalysis performed by the Dsr) was only observed in breads fermented with the LAB strains (Supplementary Figures 5A,B).

Taking into account the quantification of the isomaltose detected by the GC-MS analysis, the results reported in Figure 6 revealed that the six strains tested produced similarly high levels of soluble (210–247 mg/100 g of bread) and total (280–310 mg/100 g of bread) dextran significantly higher than those present in the control sample (34 and 80 mg/100 g of bread, respectively, with *p* = 7.58 × 10^–5^ and *p* = 3.80 × 10^–5^) (Figures 6A,B, respectively), and with no differences between each couple of parental and mutant strains (Supplementary Figures 7A,B). Therefore, the overall results obtained here support the potential usage of the *W. cibaria* riboflavin-overproducing strains to generate bread enriched in riboflavin and dextran.

In this context, the use of *W. confusa* has been reported as a promising strategy for efficient *in situ* production of dextrans in sourdoughs without strong acidification resulting in bread with improved volume and crumb softness (25). Similarly, dextran synthesized *in situ* by *W. confusa* influenced the rheological, technological and nutritional properties of whole grain pearl millet bread, leading to increased free phenolic content and antioxidant activity, as well as lowered glycemic index and improved *in vitro* protein digestibility (60). In a recent study, a mixed fermentation with *W. confusa* and *Propionibacterium freudenreichii* has been proposed for *in situ* fortification of soya flour and rice bran in order to improve texture and vitamin B_12_ content of bread (61).

The use of dextran is not widely spread in the bakery field even though its impact on bread volume and texture was shown (62). Strains of *Weissella cibaria* used as starter cultures for wheat and sorghum sourdoughs synthesized EPS (0.08 to 0.8%) and enhanced the texture, nutritional value, shelf life, and machinability of wheat, rye, and gluten-free breads (28). The addition to the dough of 10% dextran-enriched sourdough as a starter, was sufficient to achieve significant increase in wheat bread volume (63). Also, the crumb hardness of sorghum, buckwheat, teff, and quinoa breads is reduced by dextrans produced *in situ* by strains of *W. cibaria* (29, 30). Dextran content in the experimental bread prepared using parental (BAL3C-5, BAL3C-7, and BAL3C-22) or riboflavin overproducing (BAL3C-5 B2, BAL3C-7 B2, and BAL3C-22 B2) strains was about 0.30%, which is within the concentration range of hydrocolloids commercially applied in bread making (22).

## Conclusion and future perspectives

As far as we know this is the first report related to the isolation of *W. cibaria* riboflavin-overproducing mutants that are able to produce high levels of dextran. Moreover, the mutants were able to produce both riboflavin and dextran, *in situ*, during dough fermentation. Therefore, the *W. cibaria* BAL3C-5 B2, BAL3C-7 B2, and BAL3C-22 B2 strains seems to have the potential to be used for the production of functional food. As a further prospect, we will investigate whether sourdoughs fermented with the LAB mutants are able to enhance the texture, nutritional value and shelf life of sourdough-breads made at a pilot plant scale.

## Data availability statement

The datasets presented in this study can be found in online repositories. The names of the repository/repositories and accession number(s) can be found below: https://www.ncbi.nlm.nih.gov/genbank/, ON677429; https://www.ncbi.nlm.nih.gov/genbank/, ON677430; https://www.ncbi.nlm.nih.gov/genbank/, ON677431; https://www.ncbi.nlm.nih.gov/genbank/, ON677432; https://www.ncbi.nlm.nih.gov/genbank/, ON677433; https://www.ncbi.nlm.nih.gov/genbank/, ON677434; https://www.ncbi.nlm.nih.gov/genbank/, ON420949; https://www.ncbi.nlm.nih.gov/genbank/, ON420950; https://www.ncbi.nlm.nih.gov/genbank/, ON420951; https://www.ncbi.nlm.nih.gov/genbank/, ON420952; https://www.ncbi.nlm.nih.gov/genbank/, ON420953; and https://www.ncbi.nlm.nih.gov/genbank/, ON420954.

## Author contributions

MT and PL: conceptualization. AH-A, MM, PR, and RC: methodology. AH-A and JR-M: software. AH-A, JR-M, MM, PR, and RC: investigation. GSp and MM: data curation. AH-A, JR-M, MM, and PR: writing—original draft preparation. GSo, GSp, and PL: writing—review and editing. GSo, GSp, MT, and PL: supervision. GSo, PL, and MT: funding acquisition. All authors read and agreed to the published version of the manuscript.

## Footnotes

1
http://www.dietandfitnesstoday.com/riboflavin-in-white-bread.php
