# Supplementary material for: Weissella cibaria riboflavin-overproducing and dextran-producing strains useful for the development of functional bread
Source: Front Nutr. 2022 Oct 4;9:978831. doi: 10.3389/fnut.2022.978831 (PMC9577222; doi:10.3389/fnut.2022.978831)
Supplement: Supplementary file 1 [file Data_Sheet_1.docx]

Supplementary Material

*Weissella cibaria* riboflavin-overproducing and dextran-producing strains useful for the development of functional bread

**Annel M. Hernández-Alcántara^1^, Rosana Chiva^2^, Maria Luz Mohedano^1^, Pasquale Russo^3^, José Angel Ruiz-Maso^1^, Gloria del Solar^1^, Giuseppe Spano^3^, Mercedes Tamame^2^, Paloma López^1^***

^1^ Centro de Investigaciones Biológicas Margarita Salas (CIB), CSIC, Ramiro de Maeztu 9, 28040 Madrid, Spain

^2^ Instituto de Biología Funcional y Genómica (IBFG), CSIC-Universidad de Salamanca, Zacarias González 2, 37007 Salamanca, Spain

^3^ Department of Agriculture Food Natural Science Engineering, University of Foggia, via Napoli 25, 71122 Foggia, Italy

**Supplementary Table S1.** Description and location of oligonucleotides used for DNA amplification and sequencing of *rib* operon and *dsr* genes

| **Amplification and sequencing of the *rib* operon** | | |
| --- | --- | --- |
| **Amplicon**  **size (bp)** | **Primers for amplification (5’-3’) Length (nt)** | **Primers for sequencing (5’-3’) Length (nt)** |
| 4045 bp | For1: TGGCCTTGCGTGATATTTCG 20  Rev1: AGCATTGTACATCCCCTCAAA 21 | For2: TTTTGGCCCCTTTACGCAG 19  For3: TATCAAGCCGCGCAACAAG 19  For4: TCCCAATCACACACCAACAAC 21  For5: CTAGTGCGACGGCGTTTGTGATT 23  Rev2: GTGGAATTTCAAATGCGCCG 20  Rev3: CTTCAATACCGTGCTTGGCT 19  Rev4: TTCACGTGCCATCCGACCATC 21 |
| **Primer walking hybridization map** | | |
| 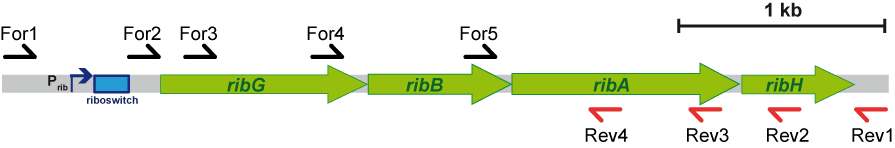 | | |
| **Amplification and sequencing of the *dsr* gene** | | |
| **Amplicon**  **size (bp)** | **Primers for amplification (5’-3’) Length (nt)** | **Primers for sequencing (5’-3’) Length (nt)** |
| 4546 bp | For1: GAAAGATTATGCCCGCGTTA 20  Rev1: GCCATATAACAGACTCCTCAAA 22 | For2: TGGCGTGAAAGTGATGGTAA 20  For3: TTGAAAATAACGGCGACACA 20  For4: TGGGTTAATGCCTACGGAAG 20  For5: CCTGCCAAATGGTATTGCTT 20  Rev2: AAAGCTTGATTGCGGACAAC 20  Rev3: CGTTGCTTACCCGTTACCAT 20  Rev4: CTACCGCACTTGCACTGTCA 20 |
| **Primer walking hybridization map** | | |
| 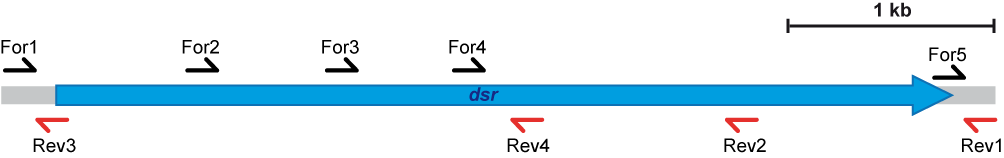 | | |

**Supplementary Table S2.** Detection of LAB survival after dough fermentation and prior to the baking process

| ***W. cibaria* strain** | **CFU/g** |
| --- | --- |
| Uninoculated LAB | 4.37 x 10^6^ |
| BAL3C-5 | 2.26 x 10^9^ |
| BAL3C-5 B2 | 1.83 x 10^9^ |
| BAL3C-7 | 1.82 x 10^9^ |
| BAL3C-7 B2 | 9.15 x 10^8^ |
| BAL3C-22 | 2.09 x 10^9^ |
| BAL3C-22 B2 | 2.81 x 10^9^ |

Doughs were independently inoculated with cells of each BAL3C strain at a concentration of 1 x 10^9^ CFU/g, and after 16 h of fermentation prior baking, the level of LAB CFU/g for the inoculated doughs was determined by plating on MRS agar medium.

To that end, 10 g samples of dough (prepared as described in Materials and Methods) were homogenized in 90 mL of sterile peptone water (1 g/L peptone, 8.5 g/L NaCl) in 250 mL flasks and incubated at 28 °C for 1 h with shaking (200 rpm). For CFU/g quantification, samples of 1 mL were collected by centrifugation at 10 000 rpm and ten-fold dilutions of the supernatants were spread as 0.1 mL aliquots on MRS agar plates that were incubated at 30 ºC for ~ 48 h.

In the spontaneously fermented uninoculated dough, low concentrations of LAB (4.37x10^6^ CFU/g), likely endogenous to the white wheat flour, were detected. In addition, values >1x10^9^ CFU/g were detected in all doughs inoculated with each BAL3C strain.

**Supplementary Table S3.** Comparison of flavin levels present in the experimental breads quantified by direct fluorescence measurement (direct determination) or after HPLC analysis (HPLC)

| ***W. cibaria* strain** | **Direct determination**  **(mg/100 g of bread)** | **HPLC**  **(mg/100 g of bread)** | **Ratio**  **Direct/HPLC** |
| --- | --- | --- | --- |
| Without BAL | 0.20±0.01 | 0.09±0.01 | 2.17 |
| BAL3C-5 | 0.24±0.02 | 0.08±0.02 | 2.90 |
| BAL3C-5 B2 | 0.45±0.04 | 0.56±0.02 | 0.81 |
| BAL3C-7 | 0.25±0.00 | 0.16±0.02 | 1.50 |
| BAL3C-7 B2 | 0.48±0.01 | 0.61±0.08 | 0.78 |
| BAL3C-22 | 0.25±0.01 | 0.13±0.02 | 1.95 |
| BAL3C-22 B2 | 0.43±0.06 | 0.46±0.06 | 0.93 |

*Bread samples were subjected to acidic and thermal treatment to convert flavins into riboflavin prior to measurement of fluorescence.


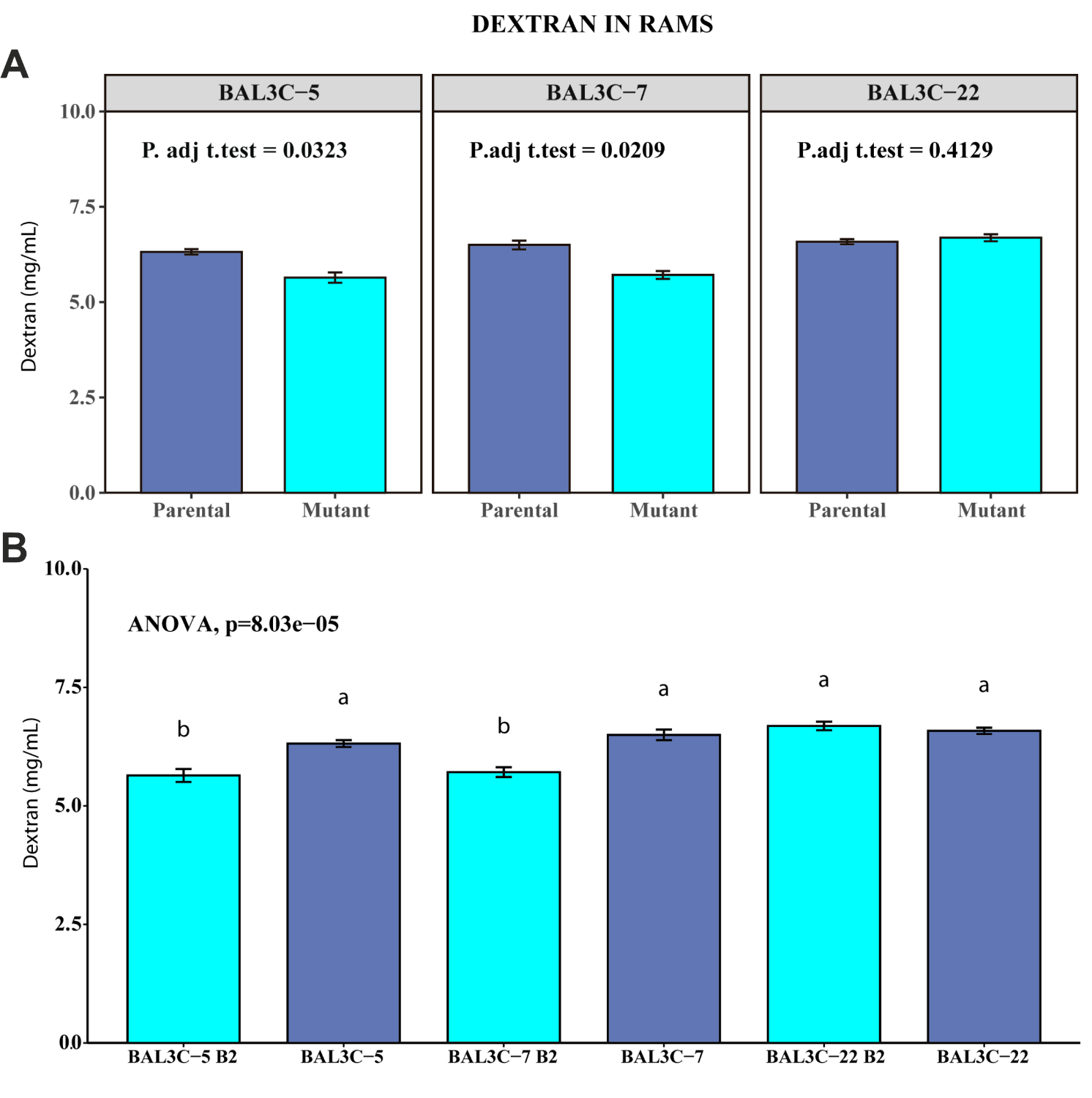


**Supplementary Figure S1.** Dextran levels produced by *W. cibaria* strains grown in RAMS medium. Values are represented as mean ± standard deviation of three independent experiments. Statistical analyses were carried out by t-test to determine if parental and mutant dextran levels were significantly different (A), or by one-way Anova to establish differences in dextran production between groups (B). In both cases a *p* value ≤ 0.05 was considered significant.

**
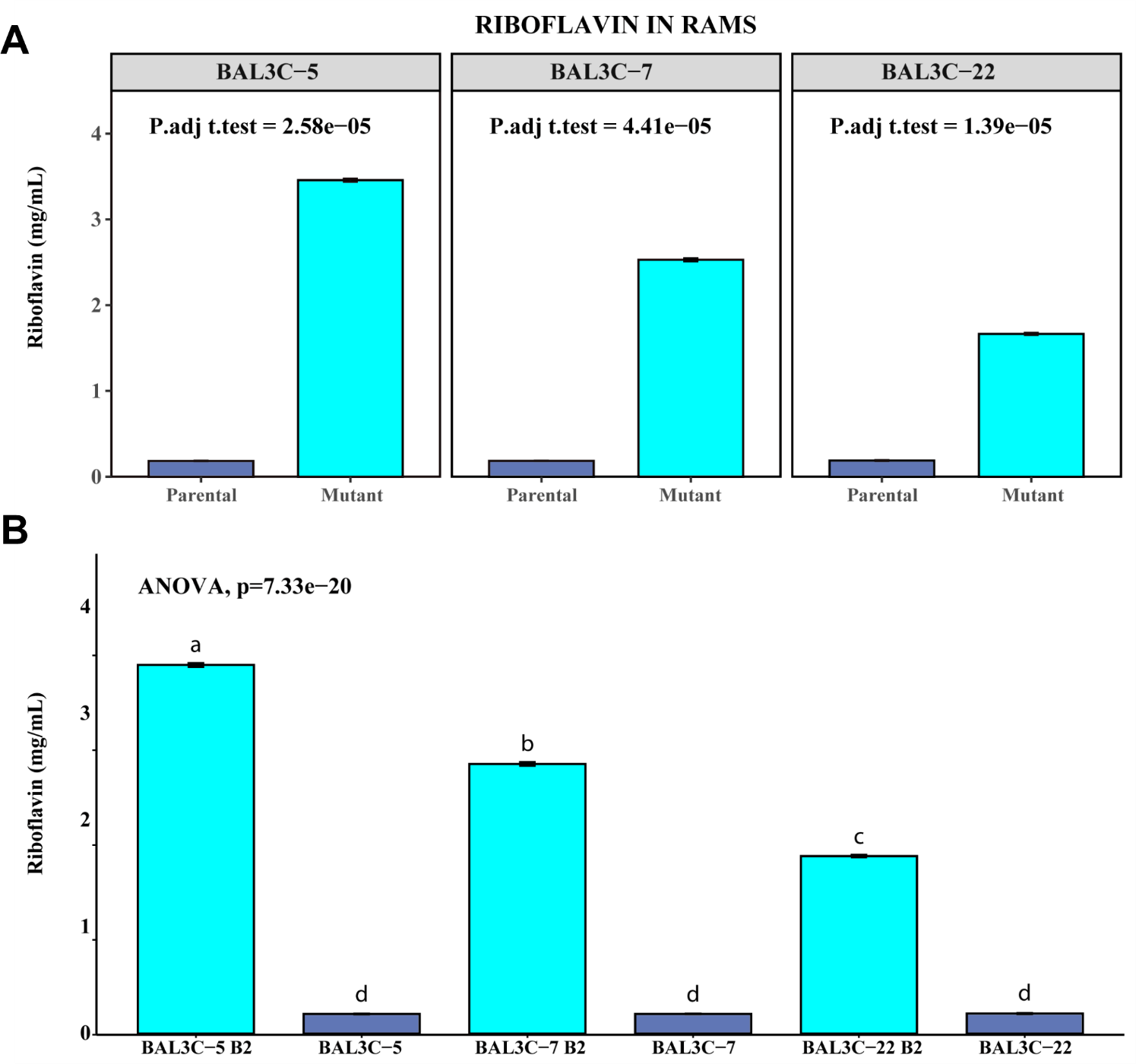
**

**Supplementary Figure S2.** Riboflavin produced by *W. cibaria* strains grown in RAMS medium. Values are represented as mean ± standard deviation of three independent experiments. Statistical analyses were carried out by t-test to determine if parental and mutant riboflavin levels were significantly different (A), or by one-way Anova to establish differences in riboflavin production between groups (B), in both cases a *p* value ≤ 0.05 was considered significant.


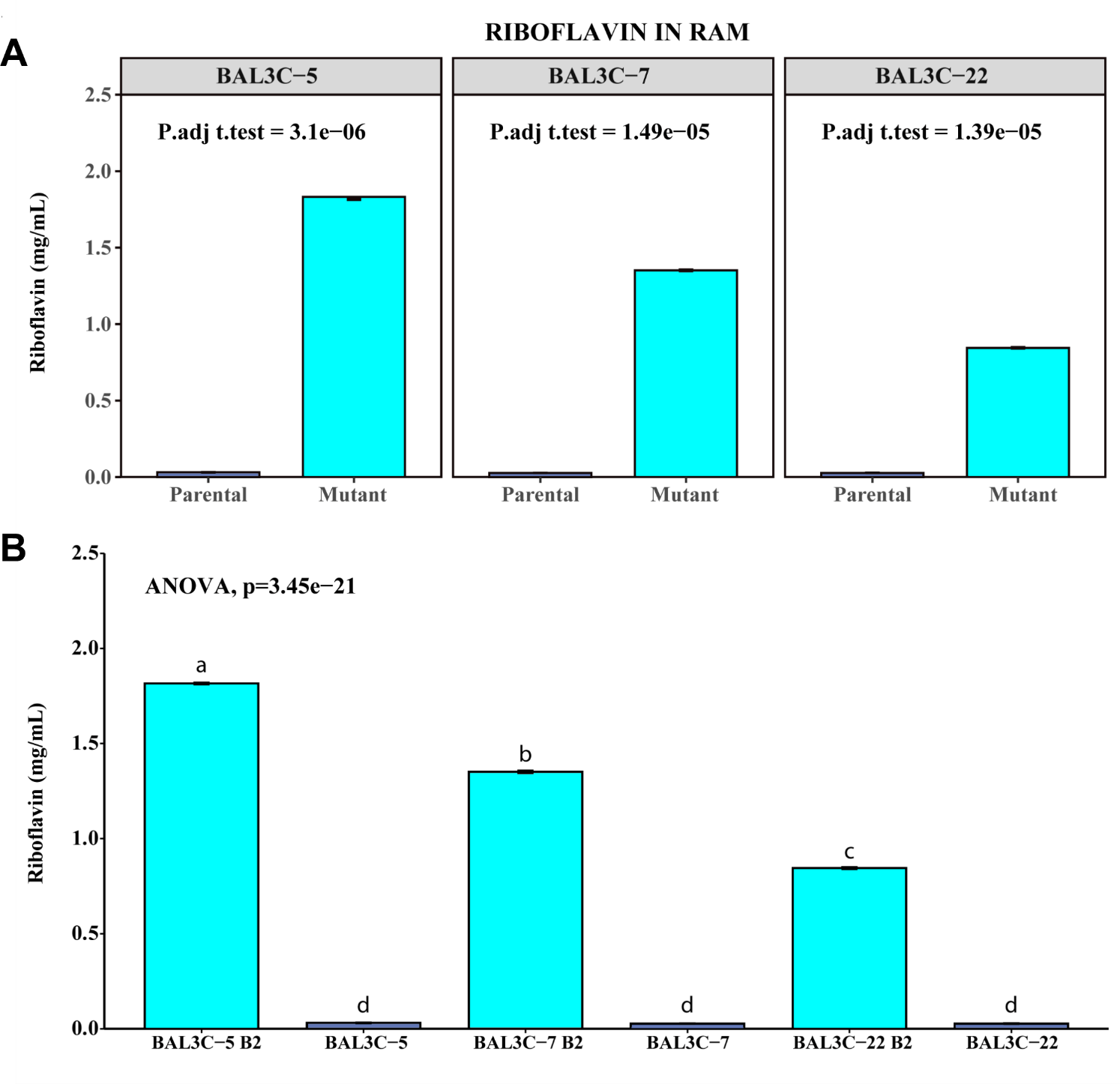


**Supplementary Figure S3.** Riboflavin produced by *W. cibaria* strains growth in RAM medium. Values are represented as the mean ± standard deviation of three independent experiments. Statistical analyses were carried out by t-test to determine if parental and mutant riboflavin levels were significantly different (A), and by one-way Anova to establish differences in riboflavin production between groups (B) in both cases a *p* value ≤ 0.05 was considered significant.

**
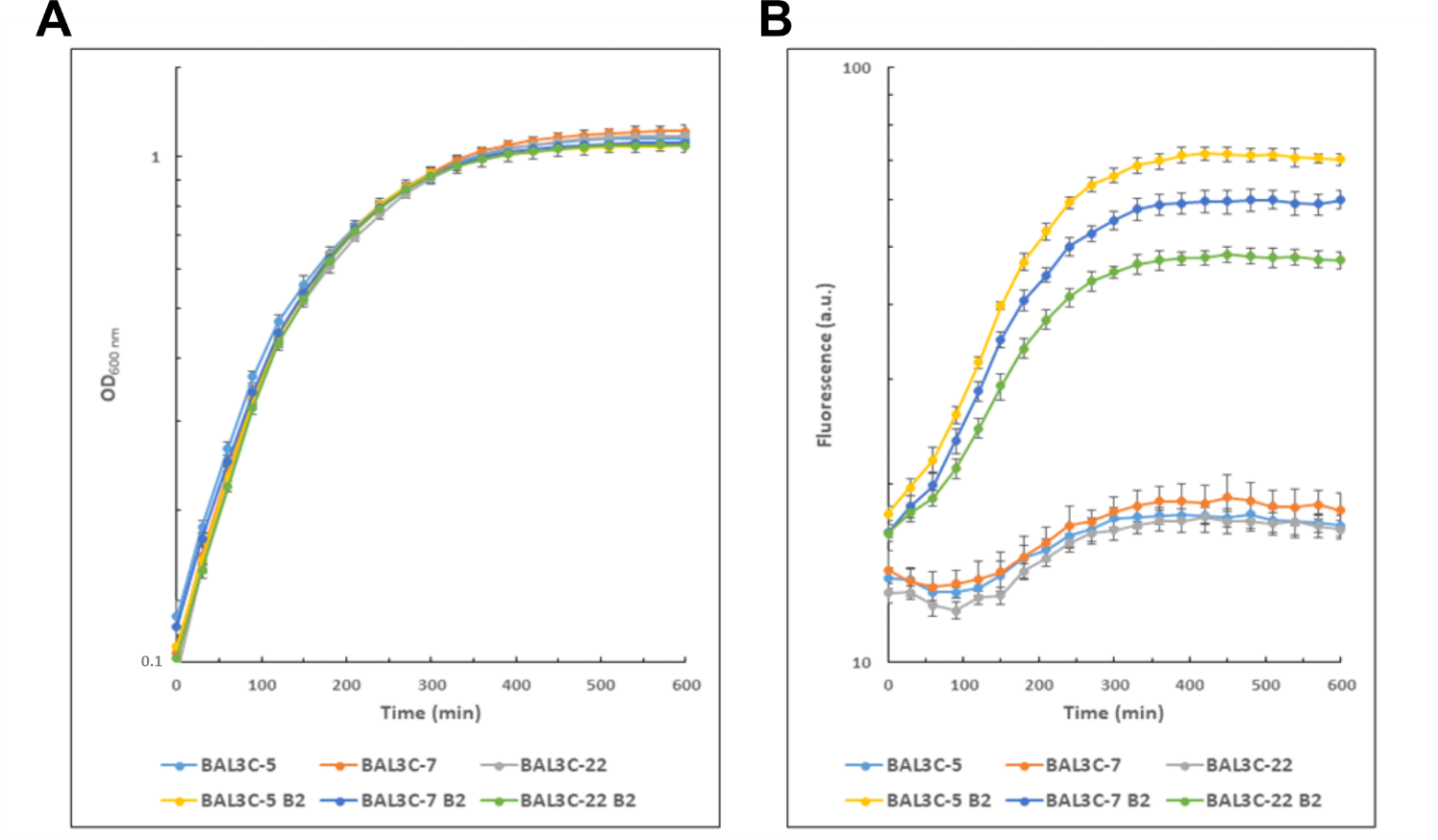
**

**Supplementary Figure S4.** Real time analysis of the influence of FMN on riboflavin production by *W. cibaria* strains. The bacteria were grown in RAMS medium supplemented with 3 μM FMN in a Varioskan Flask System. The growth was estimated by measurement of the OD_600 nm_ (A) and flavin fluorescence (B) was measured upon excitation at a wavelength of 440 nm and detection of emission at a wavelength of 520 nm.

**
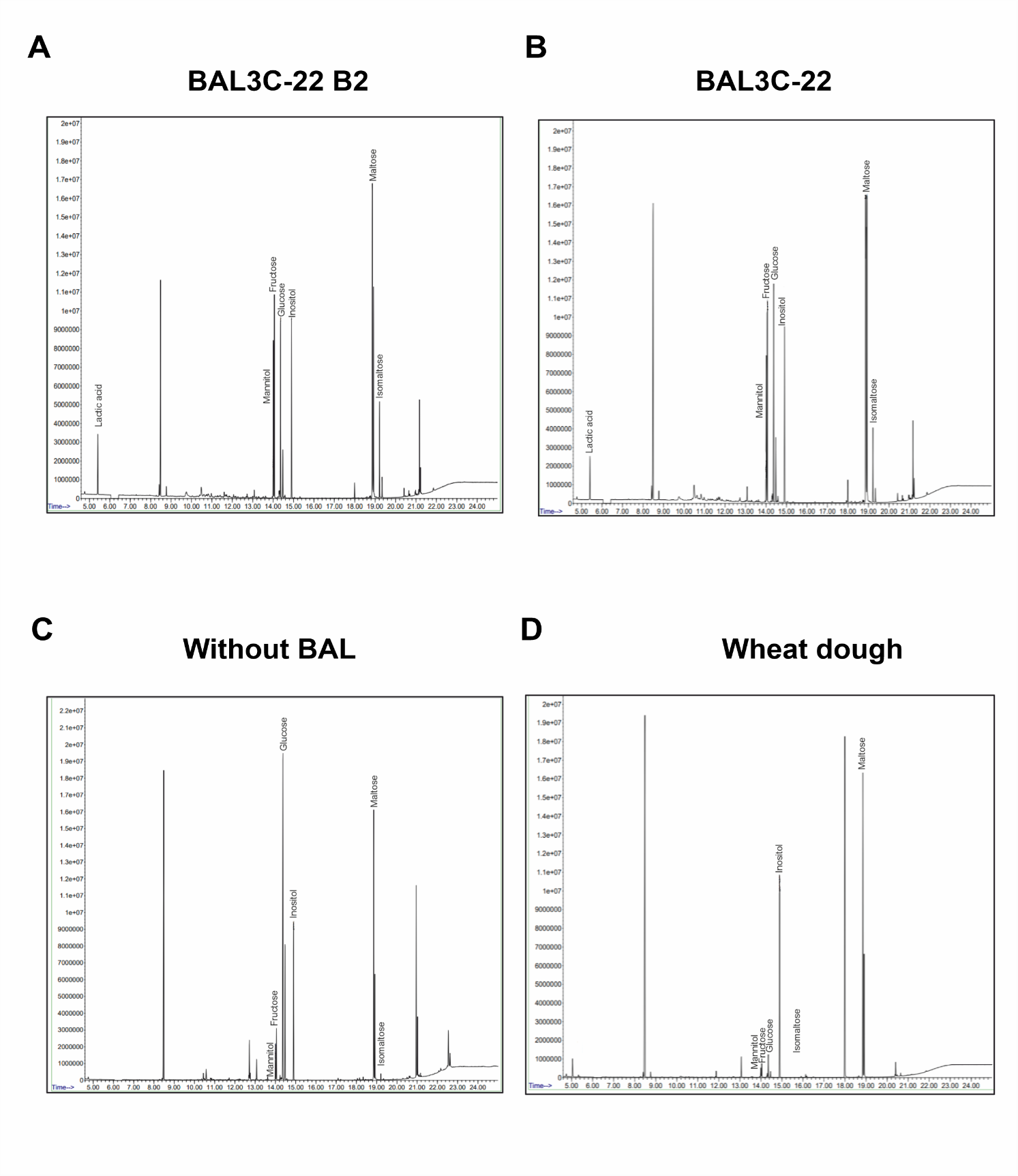
**

**Supplementary Figure S5.** GC-MS analysis of breads for detection of soluble dextran hydrolyzed to isomaltose. Chromatograms of breads produced by fermentation with BAL3C-22 B2 (A), BAL3C-22 B2 (B), with only the dough microbiota (without LAB) (C) and of wheat dough (D) are depicted. The samples were resuspended in H_2_O and treated with the *Chaetomium erraticum* dextranase at 30 °C for 18 h, as described in Material and Methods, prior to the GC-MS analysis.


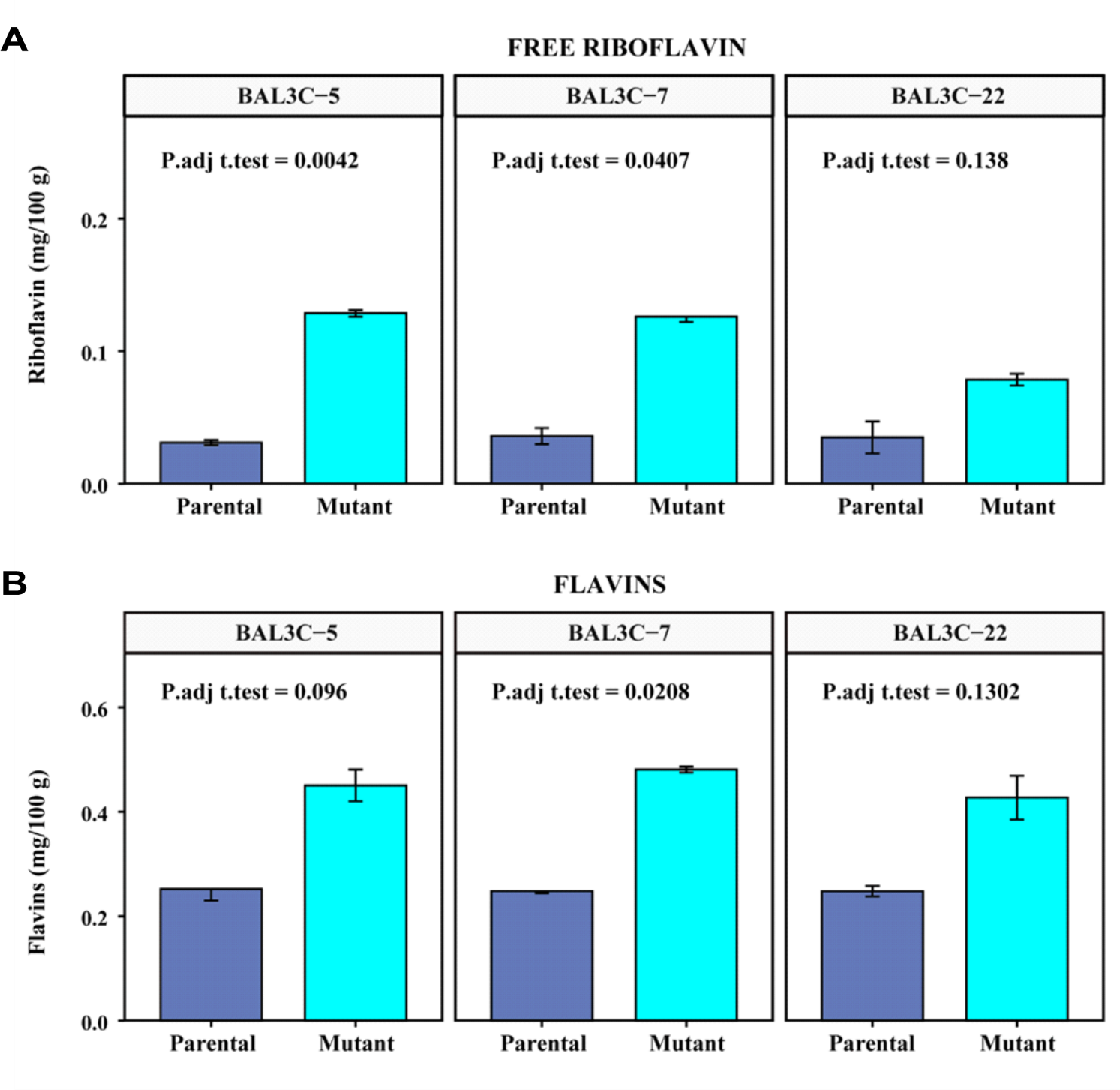


**Supplementary Figure S6.** Statistical analysis of free riboflavin (A) and flavins (B) levels in experimental breads produced with *W. cibaria* strains. Values are represented as mean ± standard deviation of three independent technical replicates. Statistical analyses were carried out by t-test to determine if levels of riboflavin and flavins synthesized by parental and mutant strains were significantly different (*p* ≤ 0.05).

**
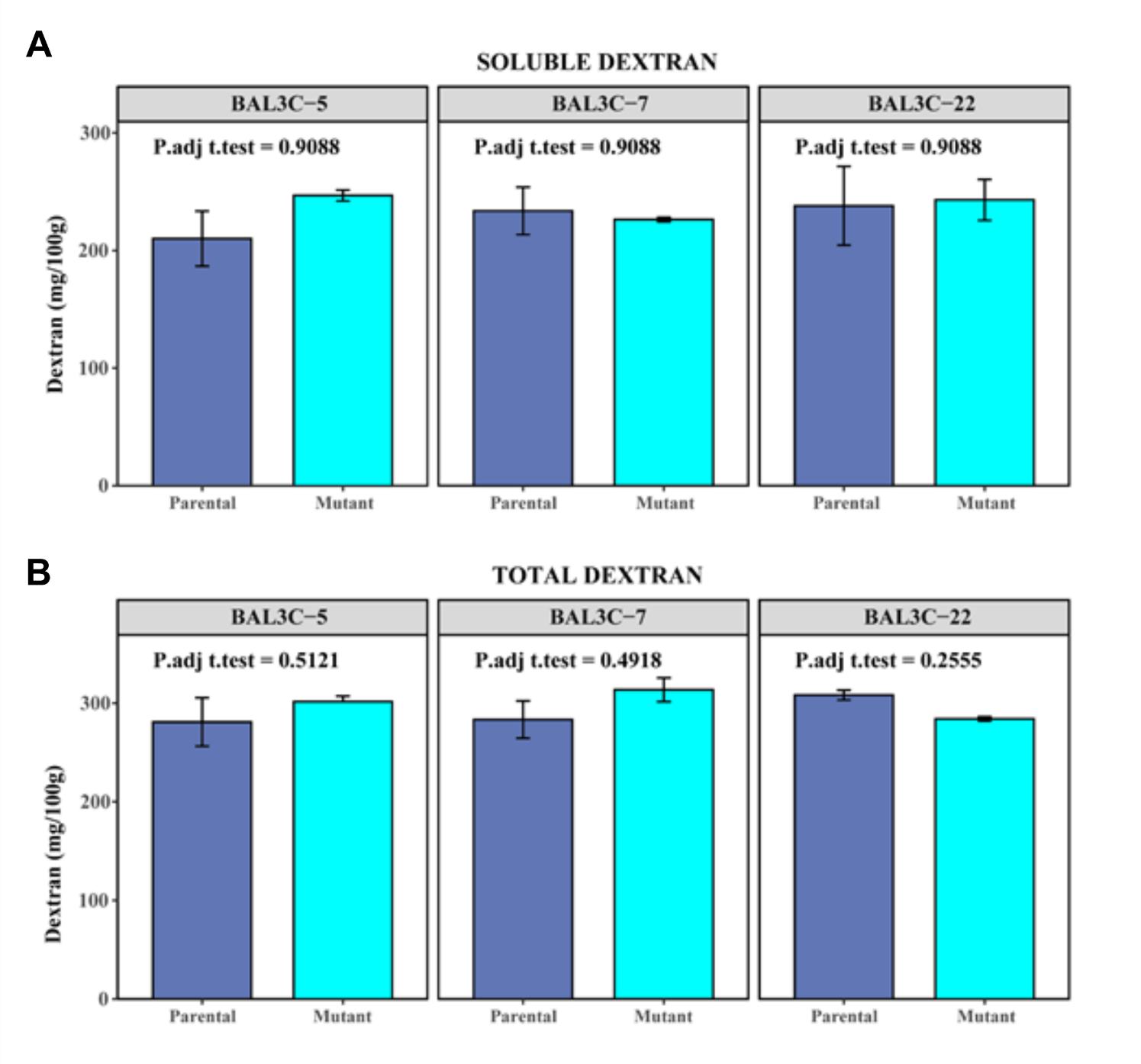
**

**Supplementary Figure S7.** Determination of soluble (A) and total (B) dextran levels in experimental breads produced with *W. cibaria* strains. Values are represented as mean ± standard deviation of three independent technical replicates. Statistical analyses were carried out by t-test to determine if levels of dextran produced by parental and mutant strains were significantly different (*p*≤ 0.05).
